# Using Fuzzy Logic in Test Case Prioritization for Regression Testing Programs with Assertions

**DOI:** 10.1155/2014/316014

**Published:** 2014-04-27

**Authors:** Ali M. Alakeel

**Affiliations:** Faculty of Computing and Information Technology, University of Tabuk, P.O. Box 1458, Tabuk 71431, Saudi Arabia

## Abstract

Program assertions have been recognized as a supporting tool during software development, testing, and maintenance. Therefore, software developers place assertions within their code in positions that are considered to be error prone or that have the potential to lead to a software crash or failure. Similar to any other software, programs with assertions must be maintained. Depending on the type of modification applied to the modified program, assertions also might have to undergo some modifications. New assertions may also be introduced in the new version of the program, while some assertions can be kept the same. This paper presents a novel approach for test case prioritization during regression testing of programs that have assertions using fuzzy logic. The main objective of this approach is to prioritize the test cases according to their estimated potential in violating a given program assertion. To develop the proposed approach, we utilize fuzzy logic techniques to estimate the effectiveness of a given test case in violating an assertion based on the history of the test cases in previous testing operations. We have conducted a case study in which the proposed approach is applied to various programs, and the results are promising compared to untreated and randomly ordered test cases.

## 1. Introduction

Program assertions have been recognized as a supporting tool during software development, testing, and maintenance [1–5]. Therefore, software developers place assertions within their code in positions that are considered to be error prone or that have the potential to lead to a software crash or failure [4]. An assertion specifies a constraint that applies to a state of computation. When an assertion is evaluated to be* false* during program execution (this scenario is called an assertion violation), there is an incorrect state in the program. Many programming languages support assertions by default, for example, Java and Perl. For languages that do not have built-in support, assertions can be added in the form of annotated statements. For example, Korel and Al-Yami [2] present assertions as commented statements that are preprocessed and converted into Pascal code before compilation. Many types of assertions can be easily generated automatically, such as boundary checks, division by zero, null pointers, and variable overflow/underflow. For this reason and to enhance their confidence in their software, programmers can be encouraged to write more assertions into their programs.

Recognizing the importance of program assertions, some recent research efforts have been devoted to the development of algorithms and methods that are specifically designed for programs that have assertions. For example, Korel et al. reported in [6] an algorithm for assertion revalidation during software maintenance. In [3], an algorithm is presented for efficient processing and analysis in which a large number of assertions are present in the program. Additionally, a regression testing method for programs with assertions was proposed in [7], and an assertion placements scheme for string-matching algorithms is presented [8].

Similar to other types of software, programs with assertions must be maintained. Software maintenance usually involves activities during which the software is modified for different reasons. Some of the reasons for which the software could be modified are fixing faults, introducing new functionality, and improving the performance of some parts of the software through the introduction of new algorithms. A study in [9] shows that there is a probability of 50–80% of introducing faults to the modified software during software maintenance. Therefore, regression testing is performed during software maintenance for the purpose of testing the modified software to ensure its correctness after maintenance operations. There are many regression testing methods, which could be classified as specification-based or code-based. Specification-based regression testing strategies, for example, [10–12], generate test cases based on the specification of the software, while code-base regression testing, for example, [7, 13–16] strategies depend on the software structural elements to generate the test cases.

Regression testing is very labor intensive and could be responsible for approximately 50% of software maintenance costs [17]. In a systematic software development environment, all of the types of regression testing methods usually involve the usage of an original test suite, which is used for the purpose of testing the original program before it has been modified. Therefore, many regression testing methods usually utilize an existing previous test suite in some form or another during regression testing. For example, a simple regression testing strategy would rerun an existing testing suite, on an as-is basis, on the modified program, while introducing new test cases to test new features. Although this method is simple, it is not practical for commercial software because an existing test suite is usually very large and could take weeks to rerun on the new modified software. Therefore, regression test selection techniques, for example, [18], test suite minimization techniques, for example, [19], and test case prioritization techniques, for example, [20–27], are proposed in the literature to mitigate the cost that is associated with running the entire suite of previous, existing tests.

The main objective of regression test selection techniques and test suite minimization techniques is to select a representative subset of the original test suite by using information about the original program, its modified version and the original test suite. It should be noted that both the regression test selection and test suite minimization techniques eliminate some elements of the original test suite, which could undermine the performance of these techniques. Test case prioritization techniques, however, order elements of the original test suite based on a given criterion. Furthermore, test case prioritization techniques do not involve the selection of a subset of the original test suite. In this presentation, we will concentrate on test case prioritization techniques; therefore, regression test selection and test suite minimization will not be discussed any further.

With regard to programs with assertions, assertions could also undergo some modifications during maintenance. Some assertions could be modified, while new assertions could also be introduced into the new version of the program. Additionally, some assertions could be kept the same as in the original program.

This paper presents a novel approach for test case prioritization during regression testing of programs with assertions using fuzzy logic. The main objective of this approach is to prioritize test cases according to their estimated potential to violate a given program assertion. Note that it has been shown in [2] that violating an assertion implies revealing a programming fault. To develop the proposed test case prioritization approach, we utilize fuzzy logic techniques [28, 29] to measure the effectiveness of a given test case in violating an assertion based on the history of the test cases in previous testing operations. The proposed method builds on previous research in the fields of assertions-based software testing and assertions revalidation, as reported in [6, 7].

The remainder of this paper is organized as follows. Related work and background is discussed in Section 2. We present our proposed fuzzy test case prioritization model in Section 3. To evaluate our proposed approach, a case study is presented in Section 4, and conclusions and future work are discussed in Section 5.

## 2. Related Work

Previous research on using fuzzy logic for the purpose of test case prioritization is scant. In [30], a fuzzy expert system is reported in which the system is used during regression testing of a telecommunication application. To build the required knowledge base for the expert system reported in this research, the researchers had to acquire knowledge from different sources, such as customer profiles, past test results, system failure rates, and the history of system architecture changes. Although this expert system has shown promising results with respect to the specific application that it was designed for, it is necessary to acquire a new knowledge base for new applications. The proposed test case selection model in [30] treats the software under test as a black box; therefore, it cannot be used for the purpose of regression testing programs with assertions.

Recently, a test case prioritization concept that is based on software agents and fuzzy logic was reported in [26]. In that research, software agents are used to gather information from different sources related to the environment surrounding the software. These sources include an architectural model, test management tool, fault management tool, and change management tool. After analyzing data that is gathered from various sources, this approach assigns each software module a test importance (TI) value in the range of 1 to 10. A high TI value indicates that this module should be tested more than another module with a lower TI value. Additionally, this approach assigns each test case a local priority (LP) value based on its ability to cover a certain software module. In the end, test cases are ordered based on global priority (GP) values, which are estimated by combining the values of the module TI values and test case LP values. The concept presented in [26] is interesting; however, the amount of data that must be gathered and analyzed by software agents could be very large and costly for large industrial software. Additionally, there has not been any information provided about what type of fuzzy logic technique has been used to estimate the TI, LP, and GP values. Furthermore, the prioritization approach reported in [5] prioritizes test cases based on their coverage ability on the module level and not on the statement level. This arrangement is a drawback because program faults are usually caused by errors at the statement level and not at the module level, which makes this approach difficult to adapt and compare with most of the existing test case prioritization methods.

### 2.1. Test Case Prioritization

The main goal of the prioritization techniques is to increase the probability of detecting faults at an earlier stage of testing [20–27]. Additionally, the test case prioritization technique objective is the utilization of previous test cases for the purpose of future testing. As stated in [21], there could exist several goals of test case prioritization, such as (1) to increase the test suite fault detection rate; (2) to minimize the time required to satisfy a testing coverage criterion; (3) to enhance a tester's confidence in the reliability of the software in a shorter time period; (4) to be able to detect risky faults as early as possible; and (5) to increase the chances of detecting faults that are related to software modification during regression testing.

In [21], an extensive study of nine different test case prioritization techniques was presented and compared according to their ability to perform fault detection during regression testing. During that study, a detection rate function is used to reorder test cases according to their ability to reveal program faults during regression testing. In [23], the Extended Finite State Machine (EFSM) system model is proposed to be used instead of real programs in order to apply the same technique presented in [24] and to reduce the cost of running test cases with real programs. Bryce et al. presented in [22] a test prioritization model for Event-Driven software. This model concentrates on testing those parts that are related to the interface in GUI applications. Several experimental results have been reported in [20], which study the cost-benefits of applying test case prioritization techniques. Recently, a method for test case prioritization using genetic algorithms was presented in [27]. In that research, a genetic algorithm is proposed to order the test cases according to their historical data with regard to their abilities to perform fault detection. A survey study of different test case prioritization techniques and mythologies has been reported in [25].

### 2.2. Regression Testing for Programs with Assertions

This section briefly introduces the concept of regression testing for programs that have assertions. For more detail, the reader is referred to [7]. Given an original program *P*_*o*_ and a modified version of this program *P*_*m*_, let *A*_*o*_ = {*a*_*o*1_, *a*_*o*2_, *a*_*o*3_, …, *a*_*on*_} be a set of assertions found in *P*_*o*_ and *A*_*m*_ = {*a*_*m*1_, *a*_*m*2_, *a*_*m*3_,…, *a*_*mz*_} be a set of assertions found in *P*_*m*_. Let *V*⊆*A*_*m*_ be a set of assertions that are nominated for revalidation [6], using previous test suites, during the process of regression testing the modified version *P*_*m*_. Depending on the type of modification that is applied to the modified version *P*_*m*_, some assertions might have been kept the same; some assertions might have been modified, and new assertions might have been introduced. The main objective of regression testing for programs that have assertions, as reported in [7], is to reduce the cost of regression testing of programs that have assertions through the utilization of previous test suites that are used during the initial development process. Furthermore, this method concentrates on assertions that are kept the same and those that are modified; new assertions are not covered because new test cases must be generated to explore these assertions. This method is presented in more detail in the next paragraph.

Let *a*_*mi*_ ∈ *A*_*m*_ be an assertion that is found in *P*_*m*_. Assume that *a*_*mi*_ was not changed from its original form in *P*_*o*_ nor was it affected by the modifications introduced to produce *P*_*m*_. Therefore, *a*_*mi*_ will be nominated by the proposed approach, to belong to the set *V*; that is, *a*_*mi*_ ∈ *V*. Suppose that assertions-oriented testing, as reported in [2], has been performed on the original version *P*_*o*_, and a set of test cases were generated during this process and were kept for later usage during regression testing. In particular, let *a*_*ok*_ ∈ *A*_*o*_ be an assertion that was found in *P*_*o*_, and let *T*(*a*_*ok*_) = {*t*_*k*1_, *t*_*k*2_, *t*_*k*3_,…, *t*_*kr*_} be the set of test cases that were generated to explore this assertion during the application of assertion-oriented testing [2] on the original program *P*_*o*_. To ensure that faults are not introduced during the production of the modified version *P*_*m*_, regression testing must be performed on *P*_*m*_, which has a set of assertions *A*_*m*_. Given *a*_*ok*_ ∈ *A*_*o*_, *T*(*a*_*ok*_) = {*t*_*k*1_, *t*_*k*2_, *t*_*k*3_,…, *t*_*kr*_} and *a*_*mi*_ ∈ *V*, it has been shown in [7] that the old test suit, *T*(*a*_*ok*_), could be used to revalidate assertion *a*_*mi*_ during regression testing of the modified version *P*_*m*_. Furthermore, it has been shown that using previous test suites to revalidate assertions could uncover faults in the modified version if these revalidated assertions were violated. More specifically, faults for which the assertions were originally designed to guard against in the original version of the program could have been reintroduced in the modified version *P*_*m*_ [7].

Although the regression testing method for programs with assertions, as presented in [7], could succeed in utilizing previous test suites and therefore reduce testing time, this method still considers using* all* test cases found in the previous test suite. Therefore, the method presented in [7] might not perform well in the presence of a large previous test suite with thousands of test cases. In this paper, we propose a test case prioritizing method that uses fuzzy logic concepts to select only a* subset* of the previous test cases. The proposed method is described in Section 3.

### 2.3. Assertions Revalidation

To address assertions in modified programs during regression testing, an assertions revalidation model was proposed in [6]. That approach is based on data dependency analysis and program slicing. In particular, that approach is based on the computation of a static slice [31, 32] for each assertion found in both the original and the modified program. These program slices are then compared to decide which assertions are to be revalidated. Although this method is very useful in identifying assertions that must be revalidated, new test cases to revalidate the assertions are generated from scratch for each assertion. For industrial size programs with a possibly large number of assertions, this approach could be very expensive.

### 2.4. Fuzzy Logic Background

In our daily life, we use words and terms that are vague or fuzzy, such as:

 “The server is* slow*,”
 “The weather is* hot*,” or
 “John is* tall*.”

Fuzzy Logic concepts, for example, [28, 29], give us the ability to quantify and reason with words that have ambiguous meanings, such as the words (*slow*,* hot*, and* tall*) mentioned above. In fuzzy sets [28], an object can* partially* belong to a set, as opposed to classical or “crisp” sets, in which an object can belong to a set or not. For example, in a universe of heights (in feet) for adult people defined as *μ* = {5,5.5,6, 6.5,7, 7.5,8}, a fuzzy subset TALL can be defined as follows:

(1) $$\begin{array}{c} \text{TALL}=\left[0/5,0.125/5.5,0.6/6,0.875/6.5,1/7,1/7.5,1/8\right]. \end{array}$$

In this example, the degree of membership for the members of the universe, *μ*, with respect to the set TALL can be interpreted as the value “6” belongs to the set TALL 60% percent of the time, while the value 8 belongs to the set TALL all of the time.

## 3. A Fuzzy Test Case Prioritization Technique

The main objective of the proposed approach in this paper is to prioritize test cases according to their effectiveness when violating an assertion. More specifically, given a set of test cases, our objective is to reorder these test cases according to their estimated potential to violate a given program assertion. Note that it has been shown in [2] that violating an assertion can strongly imply uncovering program faults.

More formally, the problem investigated in this research can be stated as follows. Given an original program *P*_*o*_ and a modified version of this program *P*_*m*_, let *A*_*o*_ = {*a*_*o*1_, *a*_*o*2_, *a*_*o*3_,…, *a*_*on*_} be a set of assertions found in *P*_*o*_, and let *A*_*m*_ = {*a*_*m*1_, *a*_*m*2_, *a*_*m*3_,…, *a*_*mz*_} be a set of assertions found in *P*_*m*_. Suppose that we are performing regression testing for the modified version *P*_*m*_, while using some regression testing method, for example, [7]. Let *T*_*o*_ = {*t*_1_, *t*_2_, *t*_3_,…, *t*_*q*_} be a previous test suite that was used during the process of assertion-oriented test data generation [2] of the original version *P*_*o*_. Given an assertion *a*_*ok*_ ∈ *A*_*o*_ and a test suite *T*(*a*_*ok*_) = {*t*_*k*1_, *t*_*k*2_, *t*_*k*3_,…, *t*_*kr*_}, which was generated to explore assertion *a*_*ok*_ during the application of assertion-oriented testing [2] on the original program *P*_*o*_. Our goal is to reorder the set *T*(*a*_*ok*_) according to the effectiveness of a given test case *t*_*kj*_ ∈ *T*(*a*_*ok*_) to violate a given program assertion *a*_*mr*_ ∈ *A*_*m*_ during the regression testing process of the modified version *P*_*m*_. We call the following effectiveness: Assertion Violating Potential (AVP) of a test case *t*_*kj*_, which is represented as AVP(*t*_*kj*_). To estimate AVP(*t*_*kj*_), we analyze the performance of each *t*_*kj*_ in previous tests of the original program *P*_*o*_ together with the revalidations [6] history of assertions found in the modified version *P*_*m*_.

**Figure 1:**
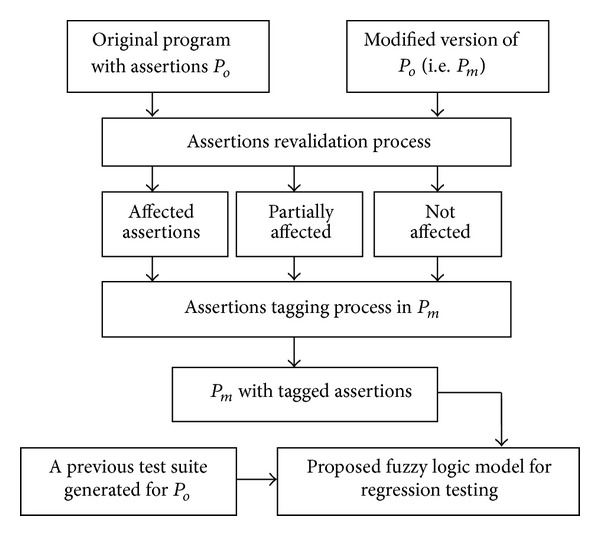
Fuzzy Regression Testing Model for programs with assertions.

We propose using the model that is shown in Figure 1, which can be described as follows. First, we analyze both *P*_*o*_ and *P*_*m*_ to classify the assertions, *A*_*m*_, that were found in *P*_*m*_ with respect to how much the modifications placed in *P*_*m*_ had affected those assertions. To perform this analysis, we use an assertions revalidations model [6] to classify the set of assertions, *A*_*m*_, which are found in *P*_*m*_ into three different sets: “Affected,” “Partially Affected,” and “Not Affected.” This categorization is based on how much each assertion has been affected by changes that were made in the modified program version *P*_*m*_. Because it is very difficult to express this categorization with normal sets that dictate drawing crisp lines between each category, we create a fuzzy set [25] called AFFECTED in which each assertion will only belong to the set by a membership value in the range [0,1]. The assignment of membership values (grades) is based on the *S*-function [33], which is shown in (4) and will be described shortly. Note that other fuzzy clustering techniques other than the *S*-function can be used for the purpose of building up fuzzy sets and the assignment of membership functions. In this research, our estimation of the modifications incurred on each assertion, *A*, is based on the number of variables modified in this assertion. More formally, let *N*_*A*_ be the number of variables that constitute an assertion *A*. Based on our empirical experiments, *S*-function parameters (*α*, *β*, and *γ* ) are expressed as follows:

(2) α=0,β=0.4∗NA,γ=0.8∗NA.

For example, if we have assertion *A* with five variables, that is, *N*_*A*_ = 5, then we will have the following *S*-function: *S*_*A*_(*x*; 0,2, 4). Based on the number of variables, *x*, modified in assertion *A*, we substitute this number in *S*_*A*_(*x*; 0,2, 4) to obtain the membership value for assertion *A*, *m*_*A*_(AFFECTED), in the fuzzy set AFFECTED. Suppose that in this example only one variable was modified in assertion *A*; the membership of *A* will be computed as *m*_*A*_(AFFECTED) = 0.125. On the other hand, if three variables were modified, then the membership of *A* will be *m*_*A*_(AFFECTED) = 0.875, and so on.

**(1):**
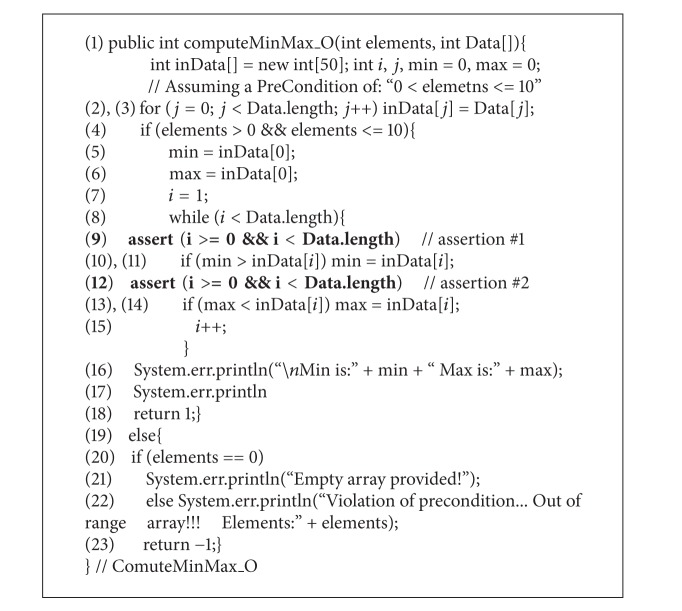
A sample Java program with assertions.

Based on the results of assertions categorization performed in the first step, the next step is to categorize test cases according to their expected effectiveness during regression testing of the modified version of the program, that is, *P*_*m*_. Because the “effectiveness” of a test case is a fuzzy term that is very hard to measure with a crisp value, we propose using fuzzy logic techniques to address measuring the effectiveness of a given test case. For this purpose, we create a fuzzy set called EFFECTIVENESS. Test cases will belong to the fuzzy set EFFECTIVENESS with a membership value or grade that corresponds to their Assertion Violating Potential (AVP) values. More specifically, let *t*_*kj*_ ∈ *T*(*a*_*ok*_) be a test case that was used to explore assertion *a*_*ok*_ ∈ *A*_*o*_ during the initial testing of a program *P*_*o*_. To measure the effectiveness of *t*_*kj*_ (with AVP(*t*_*kj*_)) in violating the corresponding assertion *a*_*mr*_ ∈ *A*_*m*_ in the modified version *P*_*m*_ during the process of regression testing the program *P*_*m*_, we use the following formula:

(3) $$\begin{array}{c} \text{AVP}\left(t_{kj}\right)=1-\text{m}{\text{a}}_{mr}\left(\text{AFFECTED}\right), \end{array}$$

where ma_*mr*_(AFFECTED) is the membership values of assertion *a*_*mr*_ in the fuzzy set AFFECTED.

Therefore, test cases related to any assertion *a*_*ok*_ ∈ *A*_*o*_, where *a*_*ok*_ belongs to the fuzzy set AFFECTED with a high membership value, will have* low* effectiveness in exploring the corresponding assertion in the modified version of the program. Similarly, test cases that are related to any assertion *a*_*ok*_ ∈ *A*_*o*_, where *a*_*ok*_ belongs to the fuzzy set AFFECTED with moderate grade values, will have* moderate* effectiveness in exploring the corresponding assertion in the modified version of the program. By the same token, test cases related to any assertion *a*_*ok*_ ∈ *A*_*o*_, where *a*_*ok*_ belongs to the fuzzy set AFFECTED with low membership values, will have* high* effectiveness in exploring the corresponding assertion in the modified version of the program.

### 3.1. The *S*-Function

*S*-functions can be described as follows [33]:

- A mathematical function that is used in fuzzy sets as a membership function.
- A simple but valuable tool in defining fuzzy functions, such as the word “*tall*”.
- The objects *x* are elements of some universe *X*. In this research, *x* represents the set of test cases that we are addressing during our prioritization mechanism, where these test cases are elements of the universe of possible program input data.
- *α*, *β*, and *γ* are parameters that can be adjusted to fit the desired membership data. The parameter *α* represents the minimum boundary, and *γ* represents the maximum boundary. The parameter *β* is the middle point between *α* and *γ* and is computed as (*α* + *γ*)/2.

The *S*-function

(4) $$\begin{array}{c} S\left(x;\alpha ,\beta ,\gamma \right)=\{\begin{array}{cc} 0 & \text{for}\;x\leq \alpha \\ 2{\left(\frac{x-\alpha }{\gamma -\alpha }\right)}^{2} & \text{for}\;\alpha \leq x\leq \beta \\ 1-2{\left(\frac{x-\alpha }{\gamma -\alpha }\right)}^{2} & \text{for}\;\beta \leq x\leq \gamma \\ 1 & \text{for}\;x\geq \gamma . \end{array} \end{array}$$

Depending on the application, a membership function can be controlled from different sources [28]. For example, in an expert system, the membership function will be constructed based on the experts' opinion modeled by the system. In this research, values of the parameters *α* and *γ* are determined after experimentation with the proposed approach.

For illustration, consider the program shown in Program 1 to be the original version *P*_*o*_, and its modified version, *P*_*m*_, is the program represented in Program 2. The function of *P*_*o*_ is to compute the minimum and maximum of an array of integers. Suppose that *P*_*o*_ is modified to introduce a new functionality, which is to compute the sum of the array elements. This modification is shown in Program 2. Furthermore, suppose that during this modification, a fault is introduced in which statement number 12 of the modified version is “incorrectly” misplaced in an incorrect position. This seeded fault will cause the program of (4) to compute the maximum element incorrectly for certain combinations of the array's elements. Note that the seeded fault could be uncovered through the violation of assertion #2, which is shown in statement number 13 of Program 2.

Using our notation above, let the identifiers “*a*_*o*2_” and “*a*_*m*2_” be used to represent assertion number 2 of Program 1 and assertion number 2 of Program 2, respectively. Note that the text of these assertions is identical in both versions of the program. Suppose that during the original application of assertion-oriented test data generation [2] on the original version of Program 1, a test suite, *A*(*a*_*o*2_), is produced during the exploration of assertion *a*_*o*2_ of Program 1. Suppose that *A*(*a*_*o*2_) is composed of five test cases, as follows:

(5) t21=(10,[17,645,−900,3,88,24,190,−10,1003,115]),t22=(10,[600,200,10000,7,99,88,42,−2000,−100,28]),t23=(10,[101,5202,700,1,32,11,270,−10,−575,9]),t24=(10,[−765,33,2009,−16,−20,113,800,19,−1,−99]),t25=(10,[−301,2045,760,10,609,24,21,−6,−14,912]).

Note that assertions-oriented testing [5] is originally proposed to be used* after* other forms of traditional software testing, such as black box (e.g., boundary value analysis) and white box (e.g., branch coverage), to increase the confidence in the software under consideration. Therefore, the test cases used in this example are only for the purpose of assertion-oriented testing [5]; hence, invalid test cases (e.g., boundary value analysis) are not included in the test suite presented above in this example.

**(2):**
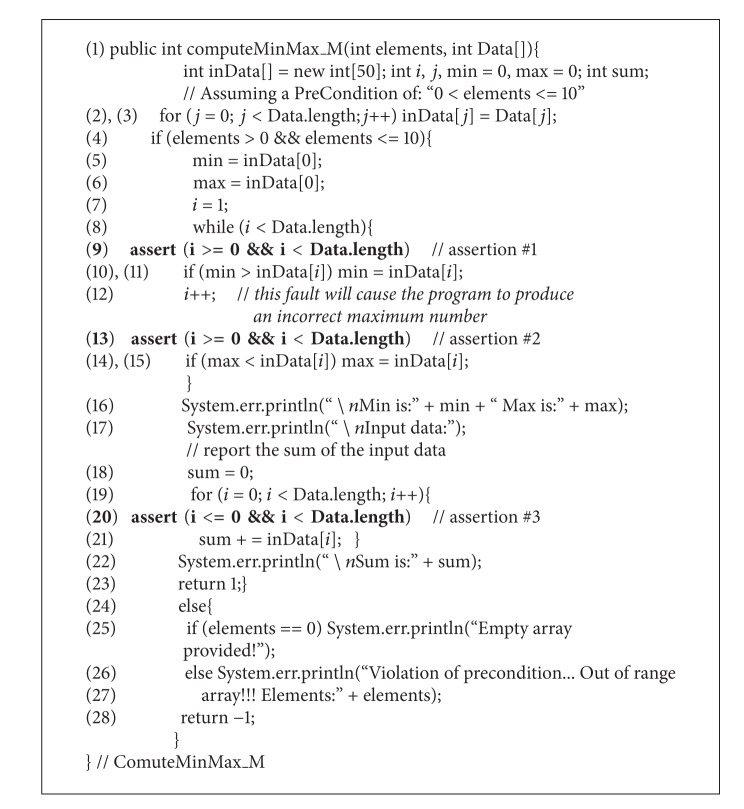
Modified version of the program in Program 1.

Note that assertions-oriented testing [5] is originally proposed to be used* after* other forms of traditional software testing, such as black box (e.g., boundary value analysis) and white box (e.g., branch coverage), to increase the confidence in the software under consideration. Therefore, test cases used in this example are only for the purpose of assertion-oriented testing [5]; hence, invalid test cases (e.g., boundary value analysis) are not included in the test suite presented above in this example.

Because assertion *a*_*m*2_ in the program of Program 2 is identical to assertion *a*_*o*2_ of the original version of Program 1, and because *a*_*m*2_ is not affected by the modifications [6] introduced to *P*_*m*_ in Program 2, the test suite generated to explore assertion *a*_*o*2_, that is, *A*(*a*_*o*2_), could be used to explore assertion *a*_*m*2_ during the regression testing of *P*_*m*_. Note that, in this example, only two test cases, *t*_23_ and *t*_25_, in *A*(*a*_*o*2_) have the potential of violating assertion number 2 of Program 2, which results in uncovering the fault in *P*_*m*_. It should be noted that assertion *a*_*m*2_ can only be violated by test cases that place the maximum element in the* second* position of the input array. As a result of these test cases, the program in Program 2 will compute the maximum element of the array incorrectly.

Applying our proposed test case prioritization approach to this example, assertion *a*_*m*2_ will be added to the set “Not Affected,” and the two test cases (*t*_23_ and *t*_25_) will be added to the fuzzy set “*High Effectiveness*.” Therefore, the proposed approach will reorder the five test cases in the test suite *A*(*a*_*o*2_), as follows: (*t*_23_, *t*_25_, *t*_22_, *t*_21_, *t*_24_). This rearrangement means that *t*_23_ and *t*_25_ will be considered first, and then, the remaining test cases are considered in random order.

## 4. Case Study

To evaluate the effectiveness of our proposed approach for test case prioritization during regression testing of programs with assertions, we have conducted an experiment in which a set of nine programs with assertions were used. These sets of programs are borrowed and are considered to be our original versions. As reported in [2], prior to assertion-oriented testing, these programs have been thoroughly tested using traditional black box testing (e.g., boundary value analysis) and white box testing (e.g., branch coverage). Additionally, from these original programs, a total of 40 new versions are created, as will be described later. Detailed information on programs used in this case study is reported in Table 1, as follows. The first and second columns show the name of the original program and the number of lines of code of this version, respectively. The number of assertions in the original program is shown in the third column, and the fourth column shows the number of modified versions created from the original program. The fifth column represents the total number of assertions in all of the versions of the same program. In the first phase of this case study, for each program from this set, we have designated a single version that we considered to be the original. For each original version, we conduct assertion-oriented test data generation as described in [2], up to a designated search time threshold of 2 minutes. During this process, we build test suites for assertions in each original program as follows.

For each assertion, we save each test case that succeeds in reaching this assertion [2, 3]. For the purpose of this experiment, the assertion's exploration process does not stop by violating an assertion—as in the original assertion-oriented testing [2]. Instead, the process of test data generation continues to produce more test cases up to a given number of violations, for example, two violations in this experiment or the exhaustion of a designated search time. The outcome of this process is a test suite for each assertion in each original program. At the end of this phase, we investigate the cause of each assertion's violation and correct faults in the original program to the best of our knowledge. In the second phase of this case study, from each original program, we create a set of modified versions. Modified versions are created by introducing three types of changes: keeping assertions the same while modifying the functionality of the new version; modifying some assertions while keeping the same functionality of the original program; and modifying some assertions and modifying the functionality of the new version. Note that new assertions could be introduced at any point in these modifications.

Additionally, and most importantly, in each modified version, we have seeded a fault that should be uncovered by an assertion's violation. In the third phase of this case study, we have identified assertions for revalidation using data dependency techniques reported in [3, 6]. In the fourth and last phase of this case study, we have conducted regression testing on all of the modified versions of each original program. During the fourth phase, previous test suites, generated during the first phase of this experiment, are used to revalidate assertions in the modified version that were identified for revalidation [6] during the third phase, and the results of this step are recorded. If a given previous test suite succeeds in reaching any assertions in the modified version, it is considered to be a success of this test suite. By reaching an assertion, we mean directing the program control flow to execute the assertion [2, 3]. If the assertion is violated, then the test suite has succeeded in uncovering the fault that was seeded in the modified version.

In this experiment, we compare the performance of the proposed “Fuzzy” test case prioritization approach to that of “Untreated” and “Random” prioritization techniques. The “Untreated” is not genuinely a technique; instead, it is used as a control. For the purpose of the untreated approach, we use the original test suites that were used for assertion-based testing of the nine original programs [7]. For all of the 40 versions of modified programs, we applied the random and fuzzy techniques to each of the 150 test suites that we created for this experiment. For the untreated, we kept the original 150 test suites because there is not any prioritization.

**Figure 2:**
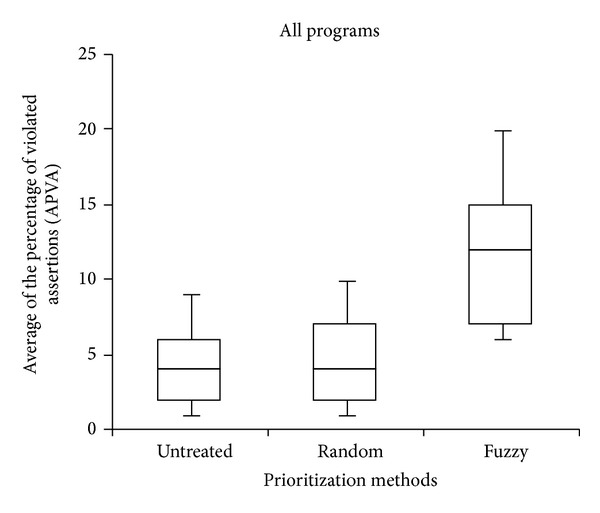
Average percentage of assertion violations achieved by each prioritization method.

For each test with a prioritized test suite, using our proposed fuzzy model, we estimated its effectiveness in violating assertions found in modified versions. More specifically, for each test suite, we measured the weighted average of the percentage of Violated assertions (WAPVA) relative to the set of assertions provided with those modified programs during the execution of a given test suite. The result of this case study is depicted in Figure 2. As shown in the box plot, a noticeable improvement in the rate of assertion violations is achieved by the proposed fuzzy prioritization approach compared to the random and untreated techniques. The random approach also produced some improvements compared to the untreated approach. Note that the maximum average percentage rate of assertion violations is approximately 25%. This finding is expected because assertion-based testing is applied only* after* traditional white-box and black-box testing techniques have been applied to the programs [2]. Therefore, the number of assertions that are expected to be violated would not be large because most program faults should have been detected by applying white-box and black-box testing techniques.

Although the result of this evaluation study is encouraging, it cannot be generalized for all types of programs with assertions. Good performance could be biased by the type of faults seeded in the programs and by the relationships among assertions found in these programs. This result, however, indicates that the proposed approach for test case prioritization enhances the effectiveness of previous test suites in violating assertions during regression testing of modified programs.

## 5. Conclusions and Future Work

In this paper, we presented a test case prioritization approach for programs that have assertions. The proposed technique employs fuzzy logic concepts to measure the effectiveness of a given test case in violating program assertions during the regression testing of modified programs. Our proposed method builds upon the concepts of previous research in the fields of assertions-based software testing and assertions revalidation. We have conducted an experimental study to evaluate the proposed approach, and the results are encouraging. Nevertheless, further investigation is still required to evaluate this approach for commercial-size software.

## Figures and Tables

**Table 1:** Programs used in the case study.

Program name	Lines of code	Number of assertions	Number of versions	Total number of assertions
Concatenation	19	4	4	20
Average	54	7	3	21
RestrictedAverage	50	6	4	30
FunnyAverage	30	5	3	15
RealNumberFormat	39	5	5	30
Bank	336	15	9	140
MinMax	23	3	6	18
Total	52	8	3	27
RestrictedSubstitute	29	3	3	10

## References

[1] Rosenblum DS Towards a method of programming with assertions.

[2] Korel B, Al-Yami AM Assertion-oriented automated test data generation.

[3] Alakeel AM (2010). An algorithm for efficient assertions-based test data generation. *Journal of Software*.

[4] Alakeel AM, Mahashi M Using assertion-based testing in string search algorithms.

[5] Alakeel AM (2010). A framework for concurrent assertion-based automated test data generation. *European Journal of Scientific Research*.

[6] Korel B, Zhang Q, Tao L Assertion-based validation of modified programs.

[7] Alakeel AM (2010). Regression testing method for programs with assertions. *American Journal of Scientific Research*.

[8] Alakeel AM Intelligent assertions placement scheme for string search algorithms.

[9] Hetzel W, Hetzel B (1991). *The Complete Guide to Software Testing*.

[10] Beydeda S, Gruhn V An integrated testing technique for component-based software.

[11] Tsai W-T, Bai X, Paul R, Yu L Scenario-based functional regression testing.

[12] Korel B, Tahat LH, Vaysburg B Model based regression test reduction using dependence analysis.

[13] Chen Y-F, Rosenblum DS, Vo K-P Test tube: a system for selective regression testing.

[14] Gupta R, Harrold M, Soffa M An approach to regression testing using slices.

[15] Korel B, Al-Yami A Automated regression test generation.

[16] Rothermel G, Harrold MJ (1997). A safe, efficient regression test selection technique. *ACM Transactions on Software Engineering and Methodology*.

[17] Beizer B (1996). *Software System Testing and Quality Assurance*.

[18] Rothermel G, Harrold MJ Safe, efficient algorithm for regression test selection.

[19] Masri W, Podgurski A, Leon D (2007). An empirical study of test case filtering techniques based on exercising information flows. *IEEE Transactions on Software Engineering*.

[20] Do H, Mirarab S, Tahvildari L, Rothermel G (2010). The effects of time constraints on test case prioritization: a series of controlled experiments. *IEEE Transactions on Software Engineering*.

[21] Rothermel G, Untcn RH, Chu C, Harrold MJ (2001). Prioritizing test cases for regression testing. *IEEE Transactions on Software Engineering*.

[22] Bryce RC, Sampath S, Memon AM (2011). Developing a single model and test prioritization strategies for event-driven software. *IEEE Transactions on Software Engineering*.

[23] Korel B, Koutsogiannakis G, Tahat LH Application of system models in regression test suite prioritization.

[24] Korel B, Tahat LH, Harman M Test prioritization using system models.

[25] Cagatay C, Mishra D (2013). Test case prioritization: a systematic mapping study. *Software Quality Journal*.

[26] Malz C, Jazdi N, Gohner P Prioritization of test cases using software agents and fuzzy logic.

[27] Huang Y-C, Peng K-L, Huang C-Y (2012). A history-based cost-cognizant test case prioritization technique in regression testing. *Journal of Systems and Software*.

[28] Zadeh LA (1965). Fuzzy sets. *Information and Control*.

[29] Kosko B (1992). *Neural Networks and Fuzzy Systems: A Dynamical Systems Approach to Machine Intelligence*.

[30] Xu Z, Gao K, Khoshgoftaar TM Application of fuzzy expert system in test case selection for system regression test.

[31] Horwitz S, Reps T, Binkley D (1990). Interprocedural slicing using dependence graphs. *ACM Transactions on Programming Languages and Systems*.

[32] Weiser M (1984). Program slicing. *IEEE Transactions on Software Engineering*.

[33] Giarratano J, Riely G (1989). *Expert Systems: Principles and Programming*.

